# Supplementary material for: Exploring the Therapeutic Potential of Elastase Inhibition in Age-Related Macular Degeneration in Mouse and Human
Source: Cells. 2023 May 3;12(9):1308. doi: 10.3390/cells12091308 (PMC10177483; doi:10.3390/cells12091308)
Supplement: Supplementary file 1 [file cells-12-01308-s001.zip › cells-2333989-supplementary.pdf]

Supplemental Methods and Materials

ELISA to detect Anti-elastin antibodies: Mouse serum elastin antibody levels were quantified with an ELISA protocol developed by us [6]. Briefly, ELISA plates were coated with 25 µg/ml mouse lung elastin peptides (Catalog# MLP54, Elastin product company) overnight at 4°C. Plates were then blocked in 0.4% milk for 1.5 h at 37°C, washed 3 times in PBST, and incubated with mouse serum at 1:100 dilution for 2 h at 37°C. Plates were again washed and incubated with biotinylated anti-mouse secondary antibodies (Goat anti-mouse IgG and Goat anti-mouse IgM-Mu chain, Vector laboratories. Inc, Burlingame, CA) overnight at 4°C followed by streptavidin incubation (cell signaling, 3999S, 1:2000 dilution) and the color development with TMB substrate (Abcam, ab171522). The reaction was stopped by adding 100 µl STOP solution (Abcam, ab171529) and the absorbance readings were taken immediately at 450 nm.

ELISA to detect serum levels of Ig antibodies: Serum Ig antibodies are measured using an Ig Isotyping ELISA kit from Invitrogen (8850660) according to the manufacturer's protocol. 10µl of serum sample was added into the wells, which were pre-coated with mouse immunoglobulin isotype-specific monoclonal antibodies conjugated to streptavidin. Plates were incubated at room temperature for 2h. TMB substrate was added for the color development and the reaction was stopped with the stop solution provided in the kit. Absorbance readings were taken immediately at 450 nm.

Sprouting assay

Six to eight months old CD1 and HTRA1 mice were sacrificed by isoflurane inhalation followed by cervical dislocation. Eyes were enucleated and placed into classic medium (Cell Systems Corporation, #50-900-036) containing supplements (Boost and Adherence Supplements) and Penicillin/Streptomycin. Connective tissue and muscles were removed and ocular pressure was released by puncturing the cornea. A cut was made below the limbus along the entire circumference of the eye, and the cornea, lens, and vitreous were removed, followed by the removal of the retina. A 1 mm wide ribbon of tissue was dissected from the margin of the posterior cup which was further cut into 1x1 mm blocks of RPE/choroid/sclera tissue [50]. Matrigel (Corning, 356231) was used undiluted at a concentration of 8.02-8.2 mg/ml. 30 µL droplets were pipetted into the wells of a 24-well plate and a piece of the tissue was placed at the center of the Matrigel dome; RPE side up. The plates were placed at 37°C for 10-20 min. To some wells, 1 µM VEGFR inhibitor Nintedanib (Tocris Bioscience, #7049) or 1 mg/mL A1AT solutions were added to the 500 µL Classic medium (control) and the plates were incubated at 5% CO_2 and 37°C for a total of 6 days with media/treatments changes every 48 hours. Tissues were fixed in 0.1% Glutaraldehyde and 2% Paraformaldehyde for 30 min at RT washed in PBS and viewed using an Olympus microscope at 4x magnification. Photographs were analyzed using Image J with macros from the Smith lab [50].

Supplemental figures and table:

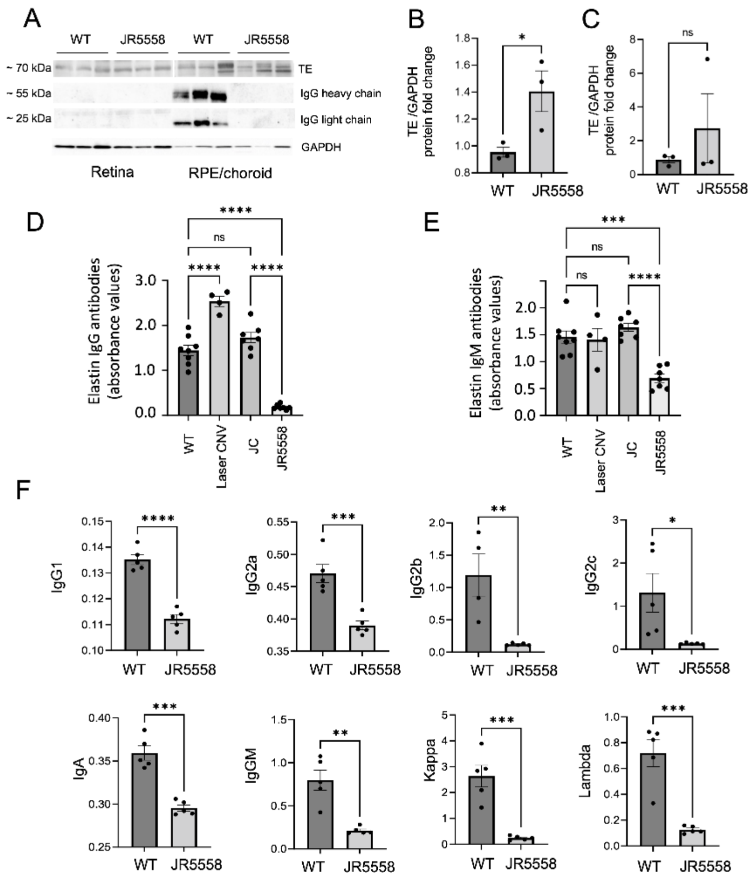

Figure S1. Elastin protein and antibody levels in mouse models of wet AMD. (A) Tropoelastin (TE) protein and total IgG antibody levels in the retinas and RPE/choroid of 4-5 months old JR and age matched WT mice (n=3 mice per group). Quantification of tropoelastin protein in the (B) retinas and (C) RPE/choroid of JR and WT mice. Serum levels of elastin (D) IgG and (E) IgM antibodies in JR, JC ($Crb1^{rd8}$ homozygous mutant mice) and laser-CNV mice compared to controls (n=4-8). (F) Quantification of serum Ig antibody levels in JR and WT (C57BL/6J) mice (n=5), Statistics used: (B, C and F) *t*-test; (D, E) one-way ANOVA, Sidak's MC, *P<0.05, **P<0.01, ***P<0.001, ****P<0.0001, ns (not significant). Data are the mean ± SEM.

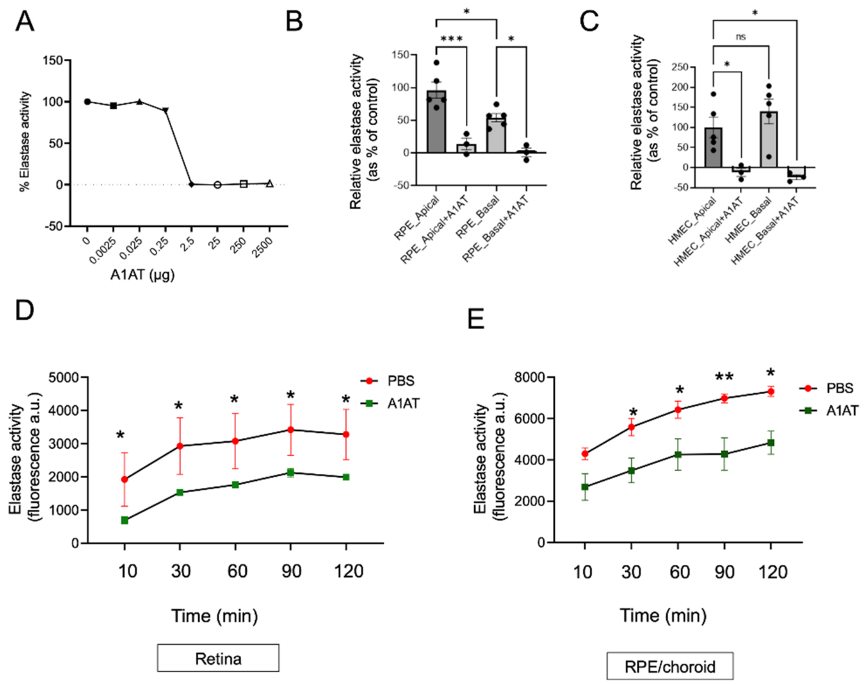

Figure S2. Alpha 1 antitrypsin (A1AT) as an elastase inhibitor. (A) In vitro elastase activity of porcine pancreatic elastase (0.025 units) is inhibited by A1AT. (B, C) Analysis of elastase activity in apical and basal supernatants of ARPE-19 and HMEC (human microvascular endothelial cell line) incubated with or without A1AT (125 µg of A1AT/per reaction well). (D) Elastase activity in retina extracts of A1AT- (7.5 mg per mouse) injected and non-injected WT mice. (E) Elastase activity in the RPE/choroid tissues of A1AT (20 mg per mouse) injected and non-injected WT mice (n=3). Statistics used: *t*-test, P<0.05, ***P<0.001, ns (not significant). Data are the mean ± SEM.

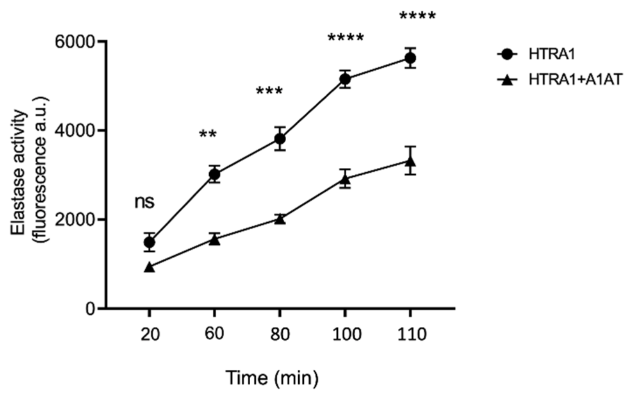

Figure S3. A1AT inhibits the elastase activity of human HTRA1. Elastase activity of 2 μg recombinant human HTRA1 protein incubated with and without 2.5 mg A1AT(n=2) Statistics used: two-way ANOVA, Sidak's MC. **P<0.01, ***P<0.001, ****P<0.0001, ns (not significant). Data are the mean ± SEM.

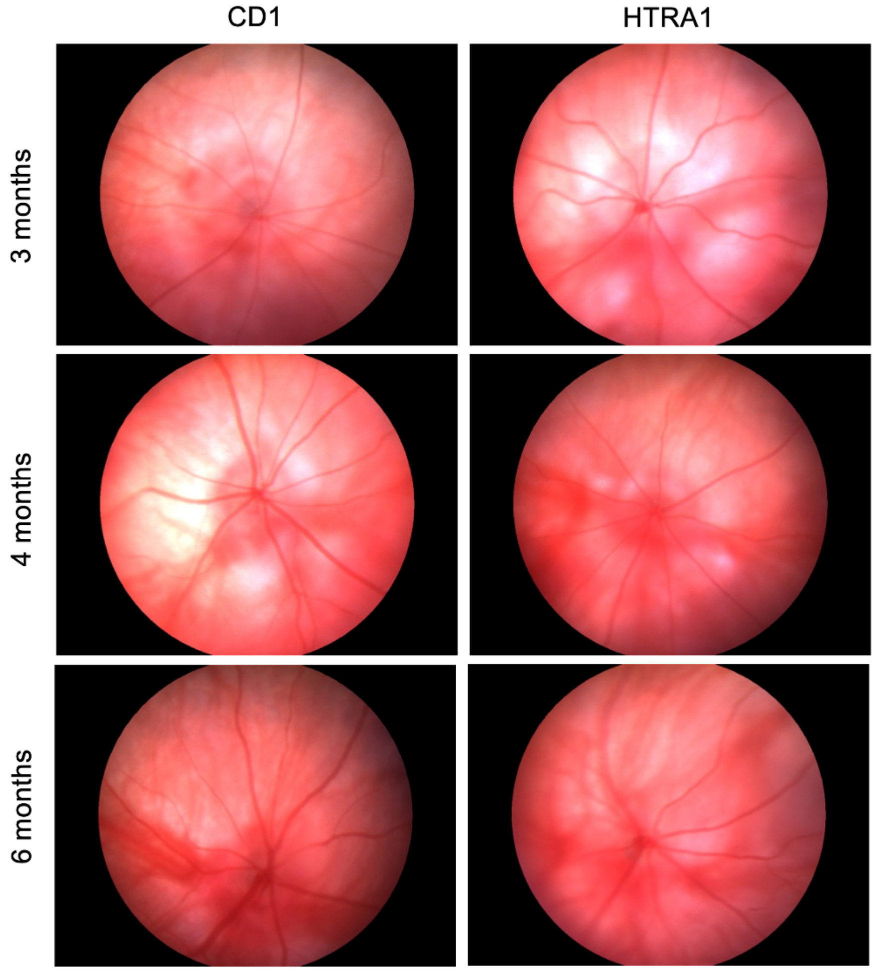

Figure S4. Fundus image analysis in HTRA1 mice. Fundus retinal images were collected at 3, 4 and 6 months of age from HTRA1 and control CD1 mice. No obvious differences were observed at any of the 3 time points. (n=3-4).

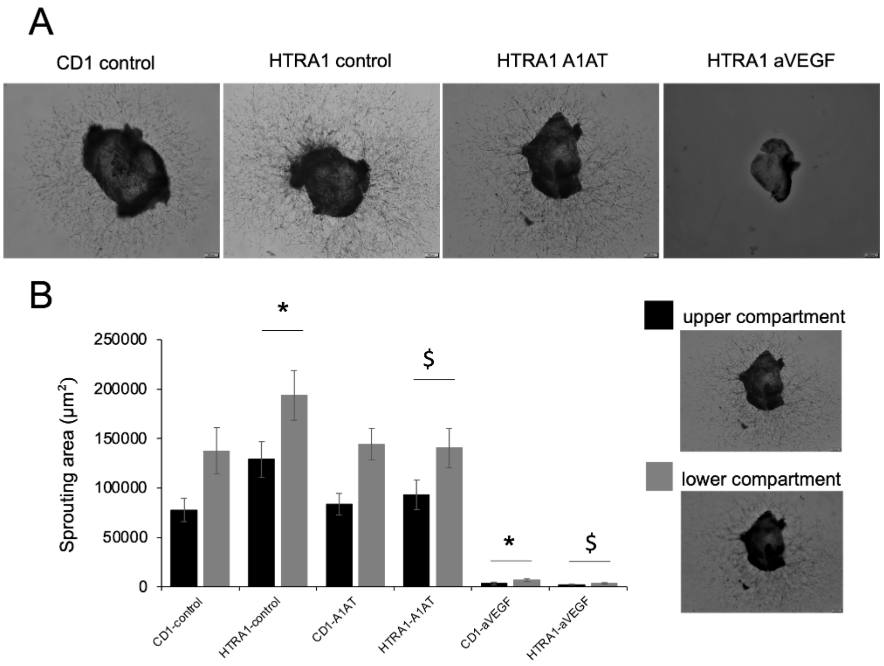

Figure S5. A1AT mitigates sprouting from RPE/choroidal tissue explants of HTRA1 transgenic mice. Outgrowth from explants represents vascular sprouts occurring in two planes. (A) Representative images of vascular sprouts from upper level. (B) Quantification of choroidal endothelial sprouting areas. Upper and lower indicates images taken from top and bottom layers of each explants (n=12-19). Statistics used: ANOVA with Fishers LSD, upper and lower values were compared as covariant – since the two growth rates are correlated. * Indicates the difference from CD1 control, $ indicates the difference from HTRA1 control, * and $ represents $P < 0.05$.

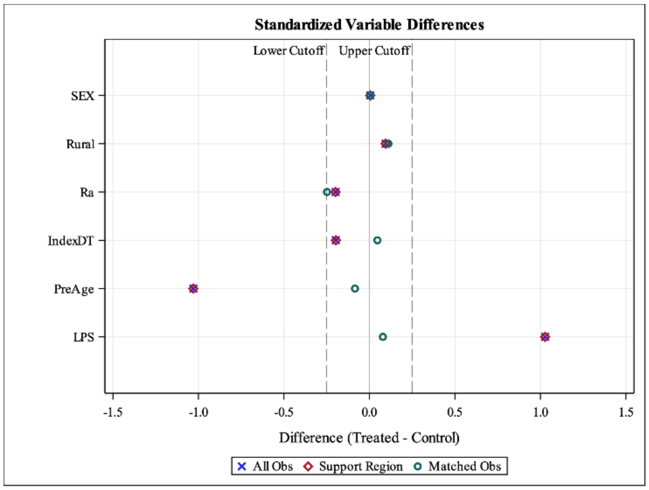

Figure S6. Standardized variable differences for A1AT-treated and control emphysema patients. For each insurance cohort we matched the A1AT-exposed patients 1:5 to the emphysema control patients using baseline variables age, sex and comorbid rheumatoid arthritis diagnosis during the baseline period. SAS Proc PSMATCH with a greedy algorithm and a caliper match of 0.2 was used. The matches resulted in two study cohorts of A1AT exposed patients and their well-matched controls, which were five times larger than the A1AT groups. All the post-match standardized differences (Green circles) fell within the prespecified cutoff region.

Variables and their Abbreviations: SEX, Rural: subjects were matched on rural versus urban as a surrogate for education, Ra: rheumatoid arthritis, IndexDT: first date observation, PreAge: baseline age, LPS: sum of all the variables.

Variable	Medicare A1AT	Medicare Controls	Commerc. A1AT	Commerc. Controls
**Number**	384	1724	1584	7900
**Mean Age (SD)**	68.2 (5.2)	68.7 (5.1)	54.5 (5.5)	54.5 (5.5)
**Percent Male**	52%	52%	48%	49%
**A1AT Exposure Years mean (SD)**	2.3 (1.9)	NA	1.9 (1.8)	NA
**Years in study Mean (SD)**	2.5 (2.0)	2.1 (1.9)	3.2 (2.7)	3.8 (2.7)

Table S1. Demographic composition and characteristics of the matched analysis cohorts. The analytical data sets were derived from screening more than 15 billion billing records for patients insured by Medicare or commercial insurance plans from 2010 through 2020. The final populations analyses were: 1) Medicare 384 patients on A1AT and 1724 controls; 2) commercially insured 1584 A1AT patients and 7900 controls.
